# Adiponectin forms a complex with atherogenic LDL and inhibits its downstream effects

**DOI:** 10.1194/jlr.RA120000767

**Published:** 2020-11-21

**Authors:** Akemi Kakino, Yoshiko Fujita, Liang-Yin Ke, Hua-Chen Chan, Ming-Hsien Tsai, Chia-Yen Dai, Chu-Huang Chen, Tatsuya Sawamura

**Affiliations:** 1 Department of Molecular Pathophysiology, School of Medicine, Shinshu University, Nagano, Japan; 2 Institute for Biomedical Sciences, Shinshu University, Nagano, Japan; 3 Department of Vascular Physiology, National Cerebral and Cardiovascular Center Research Institute, Osaka, Japan; 4 Department of Molecular Pathophysiology, Graduate School of Pharmaceutical Sciences, Osaka University, Osaka, Japan; 5 Department of Medical Laboratory Science and Biotechnology, College of Health Sciences, Kaohsiung Medical University, Kaohsiung, Taiwan; 6 Center for Lipid Biosciences, Kaohsiung Medical University Hospital, Kaohsiung Medical University, Kaohsiung, Taiwan; 7 Department of Child Care, College of Humanities and Social Sciences, National Pingtung University of Science and Technology, Pingtung, Taiwan; 8 Department of Medicine, Kaohsiung Medical University Hospital, Kaohsiung Medical University, Kaohsiung, Taiwan; 9 Lipid Science and Aging Research Center, Kaohsiung Medical University, Kaohsiung, Taiwan; 10 Vascular and Medicinal Research, Texas Heart Institute, Houston, TX, USA; 11 Graduate Institute of Medicine, College of Medicine, Kaohsiung Medical University, Kaohsiung, Taiwan

**Keywords:** cell biology, oxidized LDL, atherosclerosis, signal transduction, lipoprotein/receptors, adipocyte, ELISA, low density lipoprotein

## Abstract

Adiponectin, an adipocyte-derived protein, has antiatherogenic and antidiabetic effects, but how it confers the atherogenic effects is not well known. To study the antiatherogenic mechanisms of adiponectin, we examined whether it interacts with atherogenic low density lipoprotein (LDL) to attenuate LDL's atherogenicity. L5, the most electronegative subfraction of LDL, induces atherogenic responses similarly to copper-oxidized LDL (oxLDL). Unlike the native LDL endocytosed via the LDL receptor, L5 and oxLDL are internalized by cells via the lectin-like oxidized LDL receptor-1 (LOX-1). Using enzyme-linked immunosorbent assays (ELISAs), we showed that adiponectin preferentially bound oxLDL but not native LDL. In Chinese hamster ovary (CHO) cells transfected with the LOX-1 or LDL receptor, adiponectin selectively inhibited the uptake of oxLDL but not of native LDL, respectively. Furthermore, adiponectin suppressed the internalization of oxLDL in human coronary artery endothelial cells (HCAECs) and THP-1-derived macrophages. Western blot analysis of human plasma showed that adiponectin was abundant in L5 but not in L1, the least electronegative subfraction of LDL. Sandwich ELISAs with anti-adiponectin and anti-apolipoprotein B antibodies confirmed the binding of adiponectin to L5 and oxLDL. In LOX-1-expressing CHO cells, adiponectin inhibited cellular responses to oxLDL and L5, including nuclear factor-κB activation and extracellular signal-regulated kinas phosphorylation. In HCAECs, adiponectin inhibited oxLDL-induced endothelin-1 secretion and extracellular signal-regulated kinase phosphorylation. Conversely, oxLDL suppressed the adiponectin-induced activation of adenosine monophosphate-activated protein kinase in COS-7 cells expressing adiponectin receptor AdipoR1. Our findings suggest that adiponectin binds and inactivates atherogenic LDL, providing novel insight into the antiatherogenic mechanisms of adiponectin.

Adiponectin is a well-known adipokine with antidiabetic and antiatherogenic properties. The circulating concentration of adiponectin is characteristically high—much higher than that of other cytokines and hormones (1). Hypoadiponectinemia, characterized by an adiponectin plasma concentration less than 4 μg/ml, has been shown to be associated with abnormal glucose and lipid metabolism and an increased risk of coronary artery disease in men without a history of cardiovascular disease (2). Furthermore, individuals with inherited hypoadiponectinemia, caused by R112C and I164T amino acid substitutions in adiponectin, are known to have an increased risk for metabolic syndrome and coronary artery disease (3, 4). Despite the well-established antiatherogenic and antidiabetic properties of adiponectin, the molecular basis for these observations and the significance of adiponectin's high plasma concentration have remained largely unknown.

The antidiabetic and antiatherogenic actions of adiponectin have been extensively studied in mice and cells. In mice, overexpressing adiponectin by introducing a transgene or adenovirus vector carrying the adiponectin gene suppresses atherosclerosis in apolipoprotein E (*ApoE*)-deficient mice (5). Furthermore, deletion of the adiponectin gene in mice leads to insulin resistance and neointimal formation in a cuff injury model (6). Double knockout mice that are deficient for both AdipoR1 and AdipoR2, which are adiponectin receptors through which adenosine monophosphate (AMP)-activated protein kinase (AMPK) and peroxisome proliferator-activated receptor-α (PPARα) are activated (7), show symptoms of insulin resistance and abnormal glucose tolerance. Moreover, the deficiency of AdipoR2 has been reported to suppress atherogenesis in *ApoE* knockout mice (8). Adiponectin has also been shown to reduce the expression of proatherogenic molecules (9).

With a molecular structure that resembles that of complement C1q, adiponectin forms various multimeric structures, namely a globular form, low-molecular-weight (LMW) trimers, middle-molecular-weight (MMW) hexamers, and high-molecular-weight (HMW) multimerized forms. Another receptor for adiponectin is T-cadherin, a glycosyl-phosphatidylinositol-anchored protein that selectively binds to the hexameric and HMW forms of adiponectin (10). Recently, an association between T-cadherin and adiponectin has been suggested to work against neointimal proliferation and atherosclerosis (11).

We have previously shown that the endogenous protein developmental endothelial locus-1 (Del-1) directly blocks the interaction between atherogenic oxidized low density lipoprotein (oxLDL) and oxLDL receptors and that the overexpression of Del-1 significantly suppresses atherogenesis in mice (12). Therefore, this finding prompted us to examine whether adiponectin works similarly to Del-1 as a direct inhibitor of the interaction between atherogenic LDL and its receptors. A number of “scavenger receptors,” including scavenger receptor-A (SR-A), CD36, and lectin-like oxidized LDL receptor-1 (LOX-1), have been cloned and shown to have the ability to mediate the proatherogenic effects of oxLDL (13, 14). In addition, increasing attention has been focused on the most negatively charged (but not necessarily oxidatively modified) subfraction of LDL isolated from circulating LDL, called L5, which exerts its atherogenic effects via LOX-1 similar to oxLDL (15). In this study, we present novel evidence showing that adiponectin interacts with atherogenic oxLDL and L5 LDL and attenuates their atherogenicity, providing new insight into the antiatherogenic mechanism of adiponectin.

## Methods

### Preparation of oxLDL and DiI-labeled oxLDL

LDL (density = 1.019–1.063 g/ml) was prepared as previously described by performing the sequential ultracentrifugation of plasma from healthy volunteers (14). Isolated LDL was oxidized with 7.5 μM CuSO_4_ for 16 h and labeled with 1,1-dioctadecyl-3,3,3,3-tetramethylindocarbocyanine perchlorate (DiIC18(3); cat. no. D282, Thermo Fisher Scientific, Waltham, MA) as described previously (14).

### L5 purification

The collection of human plasma for LDL preparation was approved by the Institutional Review Board of Kaohsiung Medical University Hospital in Taiwan. Plasma LDL was isolated from pooled plasma from 9-10 patients with metabolic syndrome as previously described by using sequential potassium bromide density-gradient ultracentrifugation (density=1.019–1.063 g/ml) and was supplemented with protease inhibitor cocktail (1% penicillin/streptomycin/neomycin mixture) and 0.5 mM EDTA (16). Isolated LDL was separated into five subfractions (L1–L5) by using an anion-exchange fast-protein liquid chromatography system (GE Healthcare, Princeton, NJ) (16).

### Recombinant protein and antibodies

Human recombinant full-length adiponectin protein (composed of the LMW, MMW, and HMW isoforms) with a C-terminal FLAG sequence (produced in HEK293 cells, cat. no. RD172023100) and the globular form of adiponectin protein with an N-terminal His-tag sequence (produced in *Escherichia coli*, cat. no. RD172112100) were purchased from BioVendor (BioVendor, LLC, Asheville, NC). The following antibodies were used: anti-adiponectin polyclonal antibody (cat. no. RD181023100; BioVendor), anti-LOX-1 (#1-1) (17), anti-p44/42 MAPK (extracellular signal-regulated kinase [Erk] 1/2) (cat. no. 9102, Cell Signaling, Danvers, MA), anti-phospho-p44/42 MAPK (Erk1/2) (Thr202/Tyr204) (cat. no. 9101, Cell Signaling), anti-AMPKα (F6; cat. no. 2793, Cell Signaling), anti-phospho-AMPKα (Thr172) (40H9; cat. no. 2535, Cell Signaling), horseradish peroxidase (HRP)-conjugated anti-rabbit IgG antibody (cat. no. NA934, GE Healthcare), donkey anti-chicken IgY (cat. no. AP194P, Millipore, Burlington, MA), sheep anti-apoB polyclonal antibody conjugated with HRP (cat. no. PP086, The Binding Site, Birmingham, UK), and anti-apoB chicken monoclonal antibody (HUC20) (18, 19). HUC20 was developed as previously described (18). Epitope mapping showed that the HUC20 binding epitope of apoB is within the B1 region (amino acids 28–217 of apoB) (19).

### Enzyme-linked immunosorbent assay

HUC20 antibody (anti-apoB monoclonal antibody; 30 μl/well) dissolved in Ca^2+^, Mg^2+^-free phosphate-buffered saline (PBS) at a concentration of 5 μg/ml was added to a 384-well plate (cat. no. 781061, Greiner Bio-One, Kremsmünster, Austria) and incubated for at least 12 h at 4°C. Then, the plate was washed three times and blocked with 3% bovine serum albumin (BSA; cat. no. A7888, Sigma-Aldrich, St. Louis, MO). Next, the indicated concentrations of native LDL, oxLDL, L1, or L5 were added to the 384-well plate and incubated for 1 h at 25°C. After three washes with PBS, the plate was incubated with the indicated concentration of human recombinant full-length or globular adiponectin for 1 h at 25°C and then washed again three times with PBS. The bound adiponectin was detected with anti-adiponectin polyclonal antibody (1:1,000) and HRP-conjugated anti-rabbit IgG antibody (1:5,000). To confirm the reactivity of HUC20 to native LDL, oxLDL, L1, and L5, the bound lipoproteins were measured by using sheep anti-apoB polyclonal antibody conjugated with HRP (1:1,000). Peroxidase activity was detected by using a TMB Peroxidase EIA Substrate Kit (cat. no. 1721067, Bio-Rad Laboratories, Inc., Hercules, CA) and was quantified by measuring the absorbance at 450 nm with a microplate reader (SpectraMax, Molecular Devices, LLC, San Jose, CA).

### Cell culture and transient transfection

COS-7 cells, a transformed cell line derived from African green monkey kidney fibroblasts, were maintained in Dulbecco's modified Eagle's medium (DMEM; cat. no. 10569044, Thermo Fisher Scientific) supplemented with 10% fetal bovine serum (FBS; cat. no. SH30396.03, GE Healthcare HyClone) and antibiotic-antimycotic (cat. no. 15240062, Thermo Fisher Scientific). For experiments to determine the roles of oxLDL receptors, COS-7 cells were transfected with expression vector for LDL receptor (LDLR), LOX-1, SR-A, or CD36 by using Lipofectamine 2000 transfection reagent (cat. no. 11668019, Thermo Fisher Scientific). Briefly, 0.06 μg of each expression vector was dissolved in 7.5 μl of Opti-MEM medium (cat. no. 31985070, Thermo Fisher Scientific), and 0.18 μl of Lipofectamine 2000 was diluted in 7.5 μl of Opti-MEM medium. Then, the two solutions were mixed and incubated for 20 min at room temperature to form DNA-Lipofectamine 2000 complexes. The mixture was added to growing COS-7 cells cultured in 25 μl of DMEM/10% FBS in 384-well plates (cat. no. 781091, Greiner Bio-One) and incubated for 6 h. Subsequently, the medium was replaced by DMEM/10% FBS. For DiI-oxLDL or DiI-LDL uptake assays, cells were incubated for 42 h before the experiment. For the analysis of AMPK phosphorylation, 0.8 μg of human AdipoR1 expression vector and 1.6 μl of Lipofectamine 2000 reagent were mixed in 100 μl of Opti-MEM medium and incubated for 20 min at room temperature to form transfection complexes. The mixture was then added to growing COS-7 cells cultured in a 24-well plate (cat. no. 353047, Corning, NY). After 6 h, the culture supernatant was replaced by DMEM/10% FBS and cultured for 18 h. Cells were then subjected to AMPK phosphorylation experiments.

Tetracycline-inducible Chinese hamster ovary (CHO-K1) cells cotransfected with human LOX-1 and angiotensin II type 1 receptor (AT_1_) (LOX-1-AT_1_-CHO cells) generated in a previous study (20) were maintained in Ham's F-12 medium (cat. no. 31765-092, Thermo Fisher Scientific). The expression of LOX-1 in LOX-1-AT_1_-CHO cells was induced by incubation with 300 ng/ml doxycycline for 24 h. Human coronary artery endothelial cells (HCAECs; cat. no. CC-2585, Lonza Group, Basel, Switzerland) were maintained in endothelial cell growth medium (EGM-2; cat. no. CC-3162, Lonza Group) and used for experiments within five passages. THP-1 cells, a human macrophage-like cell line, were maintained in RPMI1940 (cat. no. 11875093, Thermo Fisher Scientific) supplemented with 10% FBS (cat. no. SH30396.03, GE Healthcare HyClone) and antibiotic-antimycotic (cat. no. 15240062, Thermo Fisher Scientific). To differentiate THP-1 cells, cells were stimulated with 100 nM phorbol 12-myristate 13-acetate for 72 h as previously described (21).

### Detection of OxLDL uptake by cells

Transfected COS-7 cells, differentiated THP-1 cells, and HCAECs were incubated with DiI-oxLDL or DiI-LDL (3 μg/ml) in the presence or absence of the indicated concentration of human recombinant full-length adiponectin or BSA in serum-free DMEM for 2 h at 37°C. To differentiate THP-1 cells, we added 100 nM phorbol 12-myristate 13-acetate to cells for 72 h as described previously (21). For the control uptake assay, adiponectin and DiI-oxLDL or DiI-LDL were added separately. Cells were initially incubated with the indicated concentration of human recombinant full-length adiponectin or BSA in serum-free medium for 2 h at 37ºC. Then, cells were washed twice with PBS and incubated with 3 μg/ml DiI-oxLDL in serum-free medium for another 2 h. After cells were washed twice with PBS, they were fixed with 10% (v/v) formalin and were counterstained with 1 μg/ml 4ʹ,6-diamidino-2-phenylindole (DAPI; cat. no. D9542, Sigma-Aldrich). Cells were subjected to fluorescence microscopy analysis (Axiovert 200M, Zeiss, Oberkochen, Germany) or quantitative fluorescence analysis by using the IN Cell Analyzer system (GE Healthcare, Chicago, IL) or Operetta (PerkinElmer, Inc., Waltham, MA).

### Western blot analysis of adiponectin in L5

LDL subfractions (2 μg of protein/lane) were denatured in 10 μl of Laemmli's sample buffer (cat. no. 161-0737, Bio-Rad) with or without 100 mM dithiothreitol and heated at 95°C for 5 min, if necessary. Proteins were separated by using sodium dodecyl sulfate polyacrylamide gel electrophoresis (SDS-PAGE, 5%–20% polyacrylamide gradient gel, cat. no. 194-15021, Fujifilm Wako Pure Chemical Corp., Osaka, Japan). Then, the proteins were electrotransferred to polyvinylidene difluoride (PVDF) membrane (iBlot2 Transfer Stacks; cat. no. IB24002, Thermo Fisher Scientific) by using an iBlot2 Dry Blotting System (Thermo Fisher Scientific). The membranes were blocked for 1 h in Immunoblock solution (cat. no. CTKN001, KAC, Kyoto, Japan) and were subsequently incubated with anti-adiponectin polyclonal antibody (1:1,000; cat. no. RD181023100, BioVendor) and anti-rabbit HRP-conjugated IgG antibody (1:5,000; cat. no. NA934, GE Healthcare). To detect apoB, we used anti-apoB monoclonal antibody (HUC20; 1 μg/ml) in conjunction with donkey anti-chicken IgY (1:4,000). To visualize immunoreactive proteins, we used an Immobilon Western Chemiluminescent HRP Substrate Kit (Millipore), and images were obtained with a chemiluminescence imager (ImageQuant LAS 4000mini, GE Healthcare).

### Luciferase reporter assay

LOX-1-AT1-CHO cells were seeded in a 96-well plate and were cotransfected with NF-κB promoter firefly luciferase reporter vector, pGF1-NF-κB (System Biosciences, Palo Alto, CA) and pRL-CMV *Renilla* luciferase control reporter vector (Promega Corp., Madison, WI) by using Lipofectamine LTX with PLUS reagent kit (cat. no. 15338100, Thermo Fisher Scientific). Briefly, in values converted to the amount per well, 100 ng of pGF1-NF-κB and 10 ng of pRL-CMV vectors were dissolved in 0.2 μl of 10 mM Tris-HCl (pH 8.0)/1 mM EDTA (TE) and added to 25 μl of Opti-MEM medium. Then, 0.1 μl of Plus reagent and 0.2 μl of Lipofectamine LTX were added to the mixture, followed by incubation for 30 min at room temperature to form DNA-Lipofectamine LTX complexes. The mixture was added to growing CHO cells cultured in 100 μl of Ham's F-12/10% FBS in 96-well plates (cat. no. 353072, Corning). After being cultured for 6 h, the cells were starved by culture in 0.1% FBS/Ham's F-12 supplemented with 300 ng/ml doxycycline to induce LOX-1 expression for 24 h. Then, oxLDL or L5 was added to cells and incubated for 20 h in the presence or absence of the indicated concentration of human recombinant full-length adiponectin or BSA. Cells were washed once with PBS and lysed by incubation with 150 μl of Passive Lysis Buffer from the Dual-Luciferase Reporter Assay Kit (cat. no. E1910, Promega) for 15 min at room temperature with mixing. Lysates (10 μl) were loaded onto a 96-well white plate (cat. no. 6002290, PerkinElmer), and firefly and *Renilla* luciferase activities were determined. Luminescence was measured by using a Powerscan four microplate reader (DS Pharma Biomedical, Suita, Japan), with 3 s of mixing time and 10 s of read time for each luciferase. Firefly luciferase activity was normalized with that of *Renilla* luciferase, and the results were expressed as the ratio of the normalized luminescence intensity of the sample to that of the control (i.e., incubated without oxLDL).

### Analysis of ERK and AMP-kinase phosphorylation

Cells were serum-starved for 24 h and treated with oxLDL and human recombinant full-length adiponectin for 5 min (to detect the ERK phosphorylation) or for 15 min (to detect the AMP-kinase phosphorylation). Then, cells were washed once with PBS and lysed by sonication with a Bioruptor (Cosmo Bio, Tokyo, Japan) in Laemmli's sample buffer (Bio-Rad) with a final concentration of 100 mM dithiothreitol. Lysates were heated at 95°C for 5 min. Proteins were separated by using SDS-PAGE and were transferred to PVDF membranes. The membranes were blocked with Immunoblock solution (KAC) and incubated with primary antibodies. The antigens were visualized by using an Immobilon Western Chemiluminescent HRP Substrate Kit (Millipore). Densitometric analysis was performed with a chemiluminescence detection system (ImageQuant LAS 4000mini; GE Healthcare).

### Measurement of endothelin-1

Endothelin-1 (ET-1) was measured as described previously (12). Briefly, HCAECs were grown in 96-well dishes (cat. no. 353072, Corning) and cultured at 37°C. Cells were serum-starved in EGM-2 for 24 h and were incubated with oxLDL (10 μg/ml) and the indicated concentration of human recombinant full-length adiponectin in 50 μl of medium for 4 h at 37°C. Conditioned medium was collected and centrifuged for 10 min at 1,500 rpm (362 *g*) by using a Centrifuge 4-15C (Qiagen, Germany), and the supernatant was collected. The concentration of ET-1 in HCAEC-conditioned medium was measured by using an ET-1 Assay Kit (cat. no. 17165, Immuno-Biological Laboratories, Gunma, Japan).

### Quantitative reverse transcriptase-polymerase chain reaction

Cells were serum-starved in EGM-2 for 24 h and treated with oxLDL (10 μg/ml) and the indicated concentration of human recombinant full-length adiponectin for 16 h. RNA isolation and cDNA synthesis were performed by using a TaqMan Gene Expression Cells-to-CT Kit (cat. no. AM1728, Thermo Fisher Scientific). Briefly, cells were washed once with PBS and incubated with 50 μl of lysis solution supplemented with DNase I, both from the kit, for 5 min at room temperature. Then, 5 μl of stop solution from the kit was added to the lysate, and the solution was incubated for 2 min at room temperature. For the reverse transcriptase (RT) reaction, 10 μl of the solution was mixed with 40 μl of RT master mix (25 μl of RT buffer, 2.5 μl of enzyme mix, and 12.5 μl of nuclease-free water) and incubated for 1 h at 37°C. The reaction mixture was heated for 5 min at 95°C to inactivate the RT. The resultant cDNA solution was then subjected to gene expression analyses. Gene expression was analyzed on an Applied Biosystems 7900HT Fast Real-Time PCR System (Applied Biosystems, Foster City, CA) by using TaqMan Gene Expression Master Mix (cat. no. 4369016, Thermo Fisher Scientific) and TaqMan Gene Expression Assays (Thermo Fisher Scientific) for monocyte chemoattractant protein-1 (MCP-1; Hs00234140m1), intercellular adhesion molecule-1 (ICAM-1; Hs99999152m1), and 18S (4319413E).

### Statistical analysis

All data are presented as the mean ± standard error of the mean. A Student's *t*-test was used for the comparison of two data sets. One-way analysis of variance and the Tukey post hoc test were used for the comparison of multiple data sets. Statistical calculations were performed in GraphPad Prism, version 7.0, for Windows (GraphPad Software Inc., San Diego, CA). A probability value (*P*) < 0.05 was considered statistically significant.

### Declaration of Helsinki

This study complied with the Declaration of Helsinki. The locally appointed ethics committee approved the research protocol, and informed consent was obtained from study participants.

## Results

### Complex formation between adiponectin and atherogenic LDL in vitro and in human plasma

**Fig. 1:**
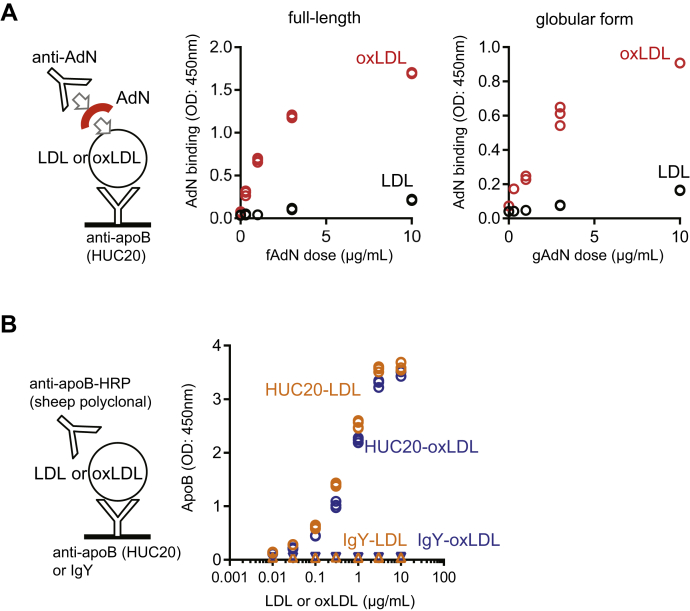
Binding of adiponectin (AdN) to oxidized LDL in vitro. A: The dose-dependent binding of AdN (human recombinant full-length or globular form) to oxLDL or LDL was detected by using an enzyme-linked immunosorbent assay (ELISA) with anti-adiponectin polyclonal antibody. Native LDL (black circles) or oxLDL (red circles) (1 μg/ml) was first captured by using an anti-apoB monoclonal antibody (ie, HUC20), and the indicated concentration of human recombinant full-length (left) or globular form (right) AdN was applied to the well. After incubation, the plate was washed, and bound recombinant human AdN was detected by using anti-AdN polyclonal antibody. B: A control ELISA with anti-apoB polyclonal antibody was performed to show that the solid-phase monoclonal antibody HUC20 (used in A) equally recognizes LDL (orange circle) and oxLDL (blue circle). As a negative control, IgY was used to instead of HUC20 (LDL, orange triangle; oxLDL, blue inverted triangle). ∗*P* < 0.05, determined by using the Tukey test. LDL, low density lipopritein; oxLDL, copper-oxidized low density lipopritein.

To examine whether oxLDL and adiponectin interact, human recombinant full-length adiponectin and the globular form of adiponectin were added to native LDL and oxLDL immobilized on anti-apoB monoclonal antibody HUC20 on the solid phase of plates (18). The extent of adiponectin binding to LDL preparations was detected by using an anti-adiponectin antibody. Both the full-length and globular forms of adiponectin were readily bound to immobilized oxLDL, whereas the binding of adiponectin to native LDL was significantly lower (Fig. 1A). The apparent Kd values were calculated as 1.41 ± 0.17 μg/ml (equivalent to 15.7 ± 1.9 nM of human full-length adiponectin trimer) for human recombinant full-length adiponectin and 2.31 ± 0.28 μg/ml (equivalent to 45.3 ± 5.5 nM of human globular adiponectin trimer) for the human recombinant globular form of adiponectin, with the assumption that the binding is 1:1 reversible binding. The anti-apoB monoclonal antibody HUC20 equally recognized native LDL and oxLDL, as confirmed by using an anti-apoB polyclonal antibody (Fig. 1B), indicating that the difference in binding between adiponectin and native LDL or oxLDL was due to the preferential recognition of oxLDL by adiponectin.

**Fig. 2:**
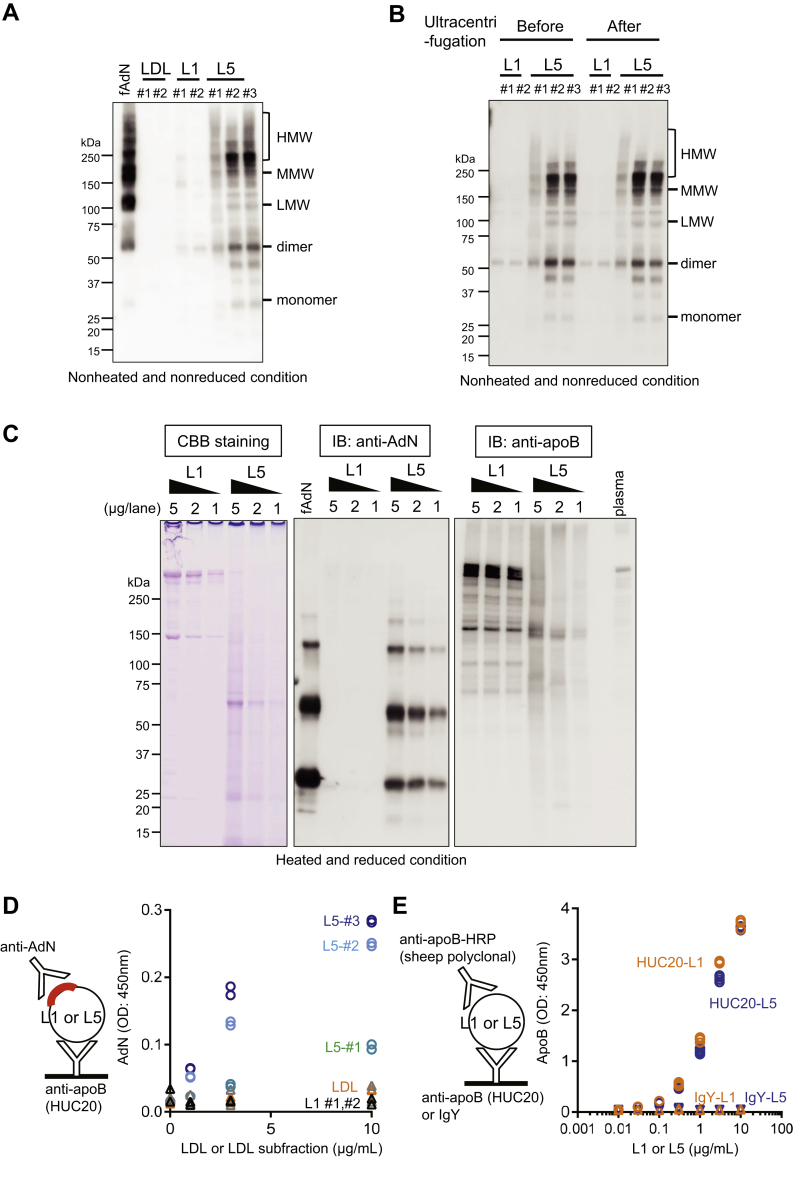
Preferential association of adiponectin (AdN) with L5 isolated from patients with metabolic syndrome. A: Immunoblotting for AdN with anti-AdN polyclonal antibody in whole LDL, L1, and L5 (2 μg each). Nonreduced and nonheated conditions were used for sodium dodecyl sulfate polyacrylamide electrophoresis. B: Immunoblotting for AdN with anti-AdN polyclonal antibody in L1 and L5 before and after a second round of ultracentrifugation to remove potentially existing exosomes. Western blotting was performed as described in (A). C: Coomassie Brilliant Blue (CBB) staining (left) and immunoblotting with anti-AdN polyclonal antibody (middle) or anti-apoB monoclonal antibody (HUC20; right) showing equal loading of L1 and L5 samples. Human recombinant full-length AdN (20 ng) was used as a positive control for the immunoblotting of AdN and was stained in parallel with L1 and L5. Human plasma (0.2 μl) was used as positive control for the immunoblotting of apoB and was stained in parallel with L1 and L5. AdN is detectable only in the L5 subfraction. The electrophoresis of samples was performed in reduced and heated conditions, so the electrophoretic pattern here is different from that in (A), (B). D: Detection of adiponectin-LDL complexes in L1 (black and gray triangles), L5 (blue, light blue, and green circles), or whole LDL (orange triangles) by using a sandwich enzyme-linked immunosorbent assay (ELISA) with anti-apoB monoclonal antibody (HUC20) and anti-AdN polyclonal antibody. E: A control ELISA with apoB polyclonal antibody was performed to confirm that the solid-phase anti-apoB monoclonal antibody (HUC20) used in (D) equally recognizes L1 (orange circles) and L5 (blue circles). As a negative control, IgY was used instead of anti-apoB monoclonal antibody (L1, orange triangles; L5, blue inverted triangles). HMW, high-molecular-weight adiponectin; LDL, low density lipopritein; LMW, low-molecular-weight adiponectin; MMW, middle-molecular-weight adiponectin.

We then explored the possibility of complex formation between endogenous atherogenic LDL and adiponectin in human plasma. We have previously demonstrated that LDL can be separated according to charge into five subfractions (L1-L5) by using anion-exchange chromatography (15). We examined the presence of adiponectin in L5, the most atherogenic and most electronegative subfraction of LDL, and in L1, the least electronegative subfraction of LDL (22). L1 is the most abundant subfraction of LDL, whereas L5 composes only 1%–2% of LDL in healthy individuals (22). Western blot analysis showed that the presence of adiponectin was prominent in L5 but hardly detectable in L1 (Fig. 2A). Because the adiponectin receptor T-cadherin has been reported to be associated with exosomes, we performed a second ultracentrifugation of L1 and L5 subfractions to remove any exosomes. After ultracentrifugation, the supernatant of L5 retained adiponectin, excluding the possibility of exosome contamination or microparticle-associated adiponectin (Fig. 2B). To confirm that the loading conditions for Western blot analysis were the same for L1 and L5, we analyzed different amounts of L1 and L5 both after electrophoresis and after Western blotting for adiponectin and apoB (Fig. 2C). Coomassie Brilliant Blue staining of electrophoresed proteins showed similar amounts of loading between L1 and L5, although L5 proteins were degraded, which is a property of L5 (Fig. 2C, left). Western blotting for apoB showed similar amounts of apoB in L1 and L5, although apoB was degraded in L5 (Fig. 2C, right). Under these conditions, adiponectin was detectable only in the lanes with L5 (Fig. 2C, middle), even when 5-fold more L1 than L5 was analyzed.

Furthermore, the results of sandwich ELISAs showed that the complex between adiponectin and L5 was simultaneously recognized by both anti-adiponectin and anti-apoB antibodies (Fig. 2D, right). This finding indicates that adiponectin and L5 are not merely cosegregated but that they are in the same molecular complex. A control ELISA with anti-apoB polyclonal antibody showed that the anti-apoB monoclonal antibody HUC20 equally recognized L1 and L5 (Fig. 2D, left), indicating that the difference in binding between L1 and L5 to adiponectin was due to the preferential recognition of L5 by adiponectin.

### Inhibition of the cellular uptake of oxLDL with physiologic concentrations of adiponectin

**Fig. 3:**
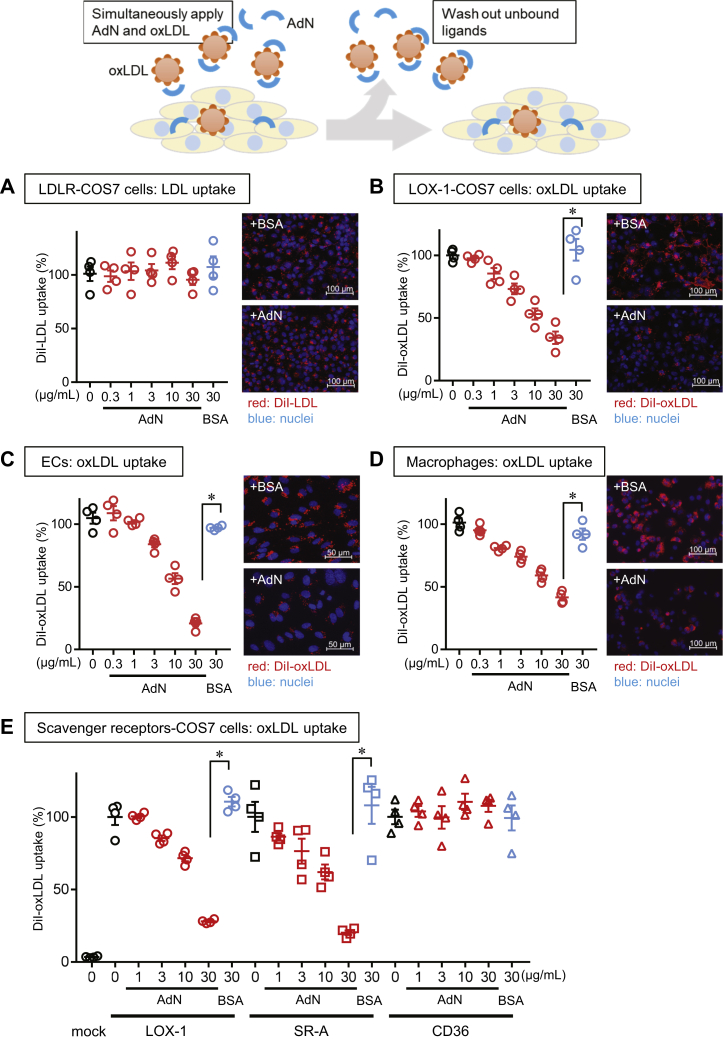
Cellular uptake of oxLDL inhibited by adiponectin (AdN) at physiologic concentrations. For the uptake assay (top schematic), the indicated cells were incubated with 3 μg/ml DiI-oxLDL (B–E) or DiI-LDL (A) in the absence (black shapes) or presence of the indicated concentration of human recombinant full-length adiponectin (red shapes) or bovine serum albumin (BSA; blue shapes). Fluorescence microscopy analysis shows DiI-LDL or DiI-oxLDL (red); cell nuclei were counterstained with DAPI (blue). A: Adiponectin had no effect on the uptake of DiI-LDL by COS-7 cells transfected with LDLR. B, C, D: In a concentration-dependent manner, adiponectin inhibited the uptake of DiI-oxLDL in COS-7 cells transfected with LOX-1 (B); in human coronary artery endothelial cells (HCAECs) (C); and in THP-1-derived macrophages (D). E: Adiponectin inhibited the uptake of oxLDL via LOX-1 or SR-A but not CD36 transfected to COS-7 cells. Data are expressed as the mean ± SEM (n = 3). Asterisks (∗) represent a significant difference (*P* < 0.05) compared with BSA at the same concentration. The DiI-oxLDL or DiI-LDL uptake values were expressed as the percentage of DiI-oxLDL or DiI-LDL fluorescence intensity of cells when incubated without AdN normalized with the number of nuclei. The fluorescence intensity of cells incubated without DiI-oxLDL or DiI-LDL was set to 0%.

We next explored how the physical interaction between adiponectin and atherogenic LDL affects the biologic activity of atherogenic LDL. When we added DiI-labeled native LDL to COS-7 cells transfected with LDLR, the presence of adiponectin (0.3–30 μg/ml) did not affect the uptake of native LDL by the transfected cells (Fig. 3A). Because LOX-1 is a receptor for oxLDL (14, 23), we examined the effect of adiponectin on the uptake of DiI-labeled oxLDL by COS-7 cells transfected with LOX-1. The addition of adiponectin (0.3–30 μg/ml) dose-dependently suppressed the uptake of oxLDL (Fig. 3B), indicating that adiponectin was selectively bound to oxLDL but not to native LDL and that the binding of adiponectin to oxLDL interfered with the receptor-mediated uptake of oxLDL in cells.

We next examined whether the inhibitory action of adiponectin on oxLDL could also be observed in endothelial cells and macrophages, which are two crucial cell types in the initiation and progression of atherosclerosis. Similar to what we observed in COS-7 cells, adiponectin suppressed the cellular uptake of DiI-labeled oxLDL in cultured HCAECs and in THP-1-derived macrophages (Fig. 3C, D).

Because various other oxLDL receptors may be expressed in these cells, we compared the effect of adiponectin on oxLDL uptake mediated by SR-A and CD36, as well as by LOX-1, in COS-7 cells (Fig. 3E). We found that adiponectin did not suppress oxLDL uptake mediated by CD36 but clearly suppressed that by SR-A, the major oxLDL receptor in macrophages that mediates foam cell formation (13). Notably, the concentration of adiponectin (10–30 μg/ml) that effectively inhibited oxLDL uptake corresponded to the physiologic concentration of plasma adiponectin.

**Fig. 4:**
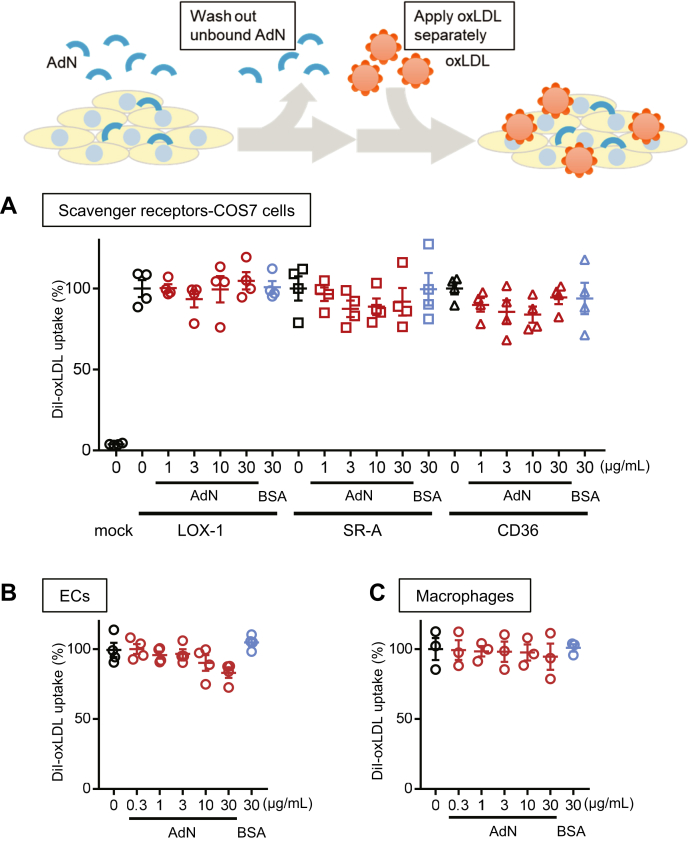
Preincubation of cells with adiponectin (AdN) did not affect the cellular uptake of oxLDL. For the uptake assay (top schematic), the indicated cells were first incubated with human recombinant full-length adiponectin or bovine serum albumin (BSA). Cells were then washed and incubated with 3 μg/mL DiI-oxLDL separately from AdN. COS-7 cells expressing LOX-1, SR-A, or CD36 (A); human coronary artery endothelial cells (HCAECs) (B); or THP-1-derived macrophages (C) were preincubated with the indicated concentration of AdN (red shapes), bovine serum albumin (BSA; blue shapes), or neither (black shapes). Then, the cells were washed, and DiI-oxLDL was added to the cells. The cellular uptake of DiI-oxLDL was not significantly changed between cells preincubated with AdN or BSA. The DiI-oxLDL uptake value was expressed as the percentage of DiI-oxLDL fluorescence intensity of cells when incubated without AdN normalized with the number of nuclei. The fluorescence intensity of cells incubated without DiI-oxLDL was set to 0%. Data are expressed as the mean ± SEM (n = 3). oxLDL, copper-oxidized low density lipopritein.

To examine the possibility that free adiponectin in the adiponectin-oxLDL mixture suppresses the cellular uptake of DiI-oxLDL, we performed experiments in which we added adiponectin and oxLDL separately (Fig. 4). After the addition of adiponectin, the cells were washed to remove any remaining adiponectin in the culture media before the addition of oxLDL. As shown in Fig. 4A, in COS-7 cells transfected with LOX-1, SR-A, or SD36, preincubation with adiponectin did not inhibit DiI-oxLDL uptake. These results represent a natural scenario because COS-7 cells do not express adiponectin receptors. Similarly, in THP-1-derived macrophages and HCAECs, preincubation with adiponectin did not inhibit DiI-oxLDL uptake (Fig. 4B, C). Careful analysis in HCAECs showed a slight trend of decreasing oxLDL uptake after preincubation with increasing amounts of adiponectin, but this trend did not reach statistical significance. Therefore, the possibility remains that, via its receptors, adiponectin inhibits oxLDL uptake to a minor extent in HCAECs.

### OxLDL-induced signal transduction inhibited by adiponectin

**Fig. 5:**
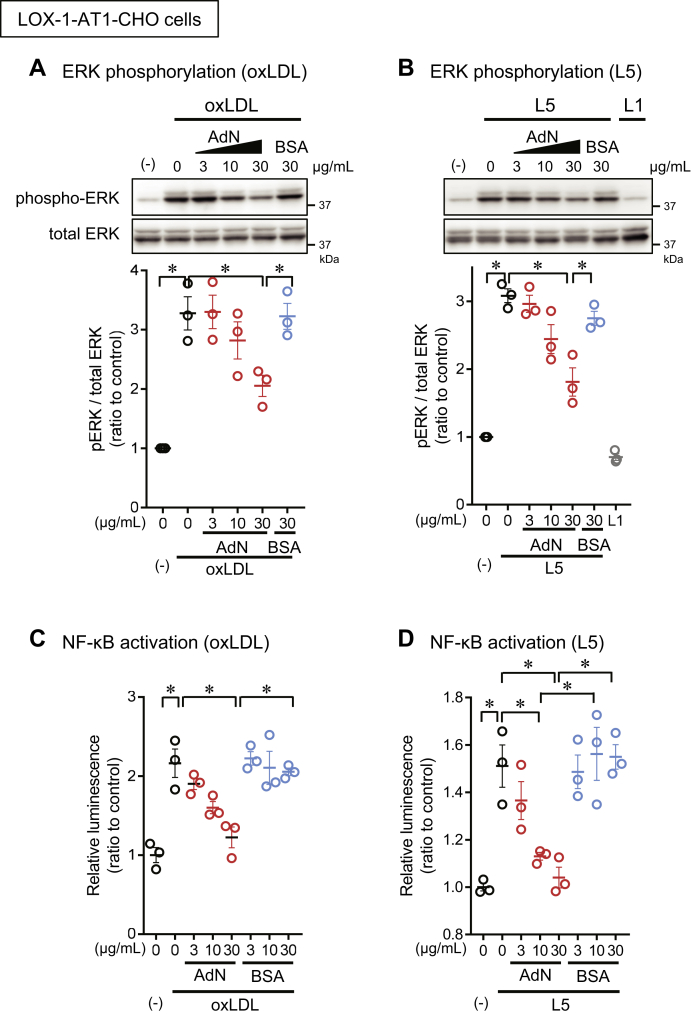
Suppression of LOX-1-mediated cellular responses to atherogenic LDL by adiponectin. A, B: Adiponectin inhibited atherogenic LDL-induced ERK phosphorylation in CHO cells expressing LOX-1 and AT_1_ (LOX-1-AT_1_-CHO cells). Cells were treated with 10 μg/ml of oxLDL (A) or L5 (B) for 5 min in the absence (black circles) or presence of the indicated concentration of human recombinant full-length adiponectin (red circles) or bovine serum albumin (BSA; blue circles). Cell extracts were subjected to Western blot analysis of phosphorylated ERK (pERK) and total ERK (42 kDa) (upper panels). The bands were quantified to calculate the ratio of pERK to total ERK (n = 3). C, D: Adiponectin suppressed atherogenic LDL-induced NF-κB activation in LOX-1-AT_1_-CHO cells. C, D: Adiponectin inhibited NF-κB activation induced by oxLDL (C) or L5 (D) in LOX-1-AT1-CHO cells. NF-κB activation was analyzed by using a luciferase reporter assay. Data are expressed as the mean ± SEM (n = 3). ∗*P* < 0.05, determined by using the Tukey test. LDL, low density lipopritein; oxLDL, copper-oxidized low density lipopritein.

**Fig. 6:**
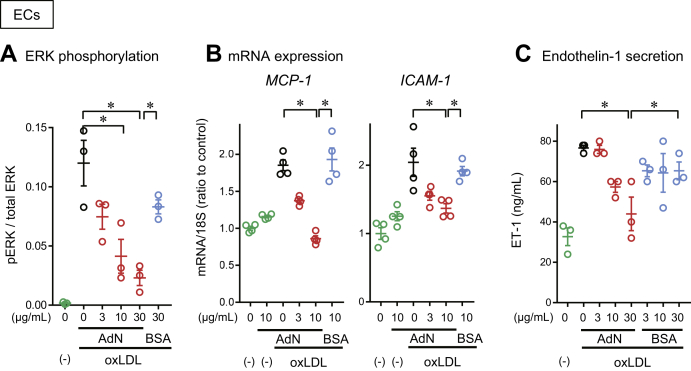
Suppression of oxLDL-induced proatherogenic responses in human coronary artery endothelial cells (HCAECs) by adiponectin. A–C: Adiponectin inhibited oxLDL-induced ERK phosphorylation (A), *MCP-1* and *ICAM-1* mRNA expression (B), and endothelin-1 secretion (C) in HCAECs. HCAECs were treated with 10 μg/ml oxLDL in the absence (black circles) or presence of the indicated concentration of human recombinant full-length adiponectin (red circles) or bovine serum albumin (BSA; blue circles). Control data from oxLDL-untreated cells are indicated with green circles. Data are expressed as the mean ± SEM (n = 3). ∗*P* < 0.05, determined by using the Tukey test. oxLDL, copper-oxidized low density lipopritein.

To confirm the inhibitory effect of adiponectin on atherogenic LDL, we analyzed the downstream signal transduction induced by oxLDL and L5 in CHO cells and endothelial cells. We found that oxLDL and L5 induced ERK phosphorylation in LOX-1-expressing CHO cells and that adiponectin dose-dependently inhibited these effects (Fig. 5A, B). Using a luciferase reporter assay, we found that oxLDL and L5 also activated NF-κB in LOX-1-expressing CHO cells and that adiponectin dose-dependently inhibited this reaction (Fig. 5C, D). Furthermore, in endothelial cells, adiponectin suppressed oxLDL-induced ERK phosphorylation (Fig. 6A), the induction of MCP-1 and ICAM-1 expression (Fig. 6B), and ET-1 secretion (Fig. 6C) in a dose-dependent manner.

### Adiponectin inhibited by oxLDL

**Fig. 7:**
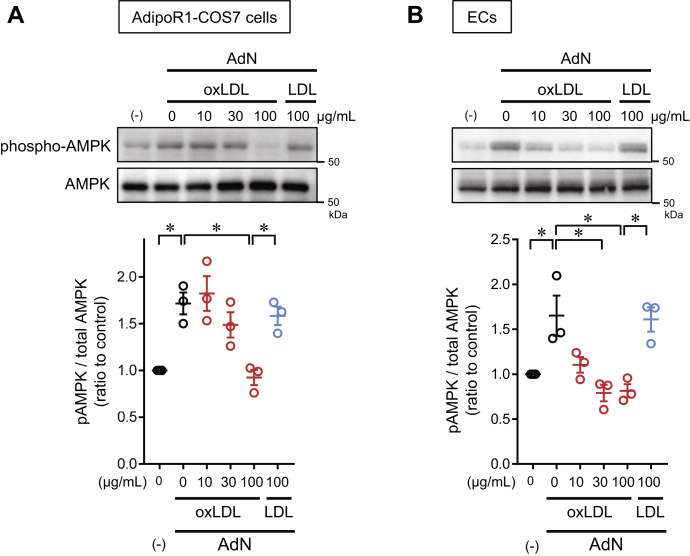
The inhibition of adiponectin-induced AMPK phosphorylation by oxLDL in AdipoR1-transfected COS-7 cells (A) and human coronary artery endothelial cells (HCAECs) (B). Cells were stimulated with human recombinant full-length adiponectin (3 μg/ml) for 15 min in the absence (black circles) or presence of the indicated concentration of oxLDL (red circles) or native LDL (blue circles). Cell extracts were subjected to Western blot analyses for phosphorylated AMPK (pAMPK) and total AMPK (62 kDa). Bands were quantified to calculate the ratio of pAMPK to total AMPK. Data are expressed as the mean ± SEM (n = 3). ∗*P* < 0.05, determined by using the Tukey test. AMPK, adenosine monophosphate-activated protein kinase; oxLDL, copper-oxidized low density lipopritein.

Last, we examined whether oxLDL conversely affects the action of adiponectin. It is known that adiponectin increases AMPK phosphorylation in endothelial cells or COS-7 cells transfected with AdipoR1 (7). When we examined the effect of oxLDL on adiponectin-induced AMPK phosphorylation, we found that oxLDL significantly inhibited adiponectin-induced AMPK phosphorylation in dose-dependent manner in both COS-7 cells and HCAECs transfected with AdipoR1, whereas native LDL had no effect (Fig. 7A, B). The more potent effects of oxLDL in HCAECs than in COS-7 cells may have been due to the combined effects of oxLDL via its receptors and its direct interaction with adiponectin in an aqueous phase.

## Discussion

In this study, our findings provide novel insight into the mechanism of adiponectin's antiatherogenic action, which may also explain the high concentration of adiponectin observed in human plasma. Although how adiponectin exerts its antiatherogenic effects has previously been poorly understood, signal transduction-mediated effects of adiponectin have been believed to play a role, such as AMP-kinase activation and the subsequent reduction of adhesion molecule expression (24). Here, we show that atherogenic LDL and adiponectin directly interact at physiologic concentration of adiponectin, which leads to the inhibition of atherogenic effects caused by oxLDL and L5, such as decreased ERK and NF-κB activation, MCP-1 and ICAM-1 expression, and ET-1 release (Fig. 6) (11).

In the general context of atherosclerosis research, showing that an endogenous molecule inhibits the action of atherogenic LDL by direct molecular interaction is an important finding. Previously, we showed that Del-1 directly interacts with oxLDL to inhibit its atherogenic action in vitro, and the overexpression of Del-1 in vivo significantly decreased the formation of atheroma induced by a high-fat diet (Paigen diet) (12). Importantly, although the overexpression of Del-1 did not alter the plasma concentration of oxLDL, it significantly decreased the receptor binding activity of oxLDL. To our knowledge, this was the first report of a protein exerting antiatherogenic effects in such a manner. However, to produce enough Del-1 to show an effect, these experiments were performed in genetically engineered mice; therefore, we could not conclude whether this mechanism operates under physiologic conditions.

Adiponectin is recognized as an adipokine but is present in human plasma at a much higher concentration (5–10 μg/ml, 50–100 nM as a homotrimer) (25) than other hormones and cytokines, such as angiotensin II (1 nM), insulin (<1 nM), and IL-6 (<1 pM). A functional explanation for this observation has been previously lacking. The phagocytosis of dead cells was shown to occur via adiponectin at concentrations similar to those found in plasma (1, 26). In addition, genetic analyses of a healthy middle-aged cohort of individuals showed that hypoadiponectinemia was associated with the risk of atherosclerosis (2, 3, 4). Notably, the binding of adiponectin to its receptors occurs at much lower concentrations than its plasma range. This suggests that the presence of adiponectin at its high physiologic concentration has an antiatherogenic role independent from its receptor-mediated properties. In the present study, adiponectin at physiologic concentrations (3–30 μg/ml) interacted with oxLDL and exerted antiatherogenic actions, providing evidence for the first time to our knowledge that adiponectin is a physiologic endogenous antagonist for oxLDL. Notably, we detected adiponectin-L5 complex formation even after the purification of L5. Figure 2 shows a complex between adiponectin and oxLDL already formed in vivo. Because L5 was purified by means of ultracentrifugation, dialysis, and anion-exchange chromatography, most of the adiponectin associated with L5 in plasma may have become dissociated during these processes, especially with the presumably low-affinity binding between adiponectin and L5 that occurs under a high physiologic concentration of adiponectin. However, even under such conditions, we still detected adiponectin in L5. Although we clearly showed that the inhibitory effect of adiponectin on oxLDL stems from oxLDL-adiponectin complex formation, it is not our intention to negate the various effects of adiponectin mediated by its receptors. Of note, Ouchi and Walsh (1) have reported that the 2-day incubation of macrophages with 30 μg/ml adiponectin reduced SR-A expression. Rather, the present study sheds light on a previously overlooked role of adiponectin that potentially occurs in an aqueous phase, such as in blood.

Despite the well-known antiatherogenic properties of adiponectin, complex in vivo phenomena related to adiponectin have been previously reported, referred to as the “adiponectin paradox.” For example, too much adiponectin has been shown to have negative effects (27). In addition, in obese individuals, adiponectin concentrations are reduced in the plasma, despite the overabundance of adipose tissue (28). Furthermore, in elderly people or cohorts with prevalent cardiovascular disease, adiponectin levels have been positively associated with all-cause/cardiovascular disease mortality (29). The reasons for this remain unknown, despite various possible explanations, including the compensatory secretion of adiponectin. “Adiponectin resistance” has also been speculated as a cause, although the basis of this is unknown (27, 30).

To help understand these phenomena, the recent advances made toward understanding the role of HDL in atherosclerosis should be considered for the sake of comparison. According to a recent epidemiologic study, an HDL level that is too high is a risk factor for cardiovascular disease, despite that HDL is well recognized as an antiatherogenic lipoprotein. HDL has also been shown to have proatherogenic properties in patients with coronary artery disease or with a high risk of coronary artery disease (31). These findings have suggested that, under such conditions, HDL becomes dysfunctional by losing its antiatherogenic properties (i.e., paraoxonase-1 activity and cholesterol efflux capacity) and gains proatherogenic properties (i.e., binding to the atherogenic receptor LOX-1) (32).

In the present study, the binding of adiponectin to atherogenic LDL also attenuated the function of adiponectin. Thus, adiponectin bound by atherogenic LDL becomes dysfunctional. It is unknown to what extent this binding contributes to the dysfunction of adiponectin under human physiologic conditions, but the cosegregation of adiponectin and atherogenic LDL in human plasma suggests that this mechanism functions in humans. This may constitute the mechanism of adiponectin resistance.

**Fig. 8:**
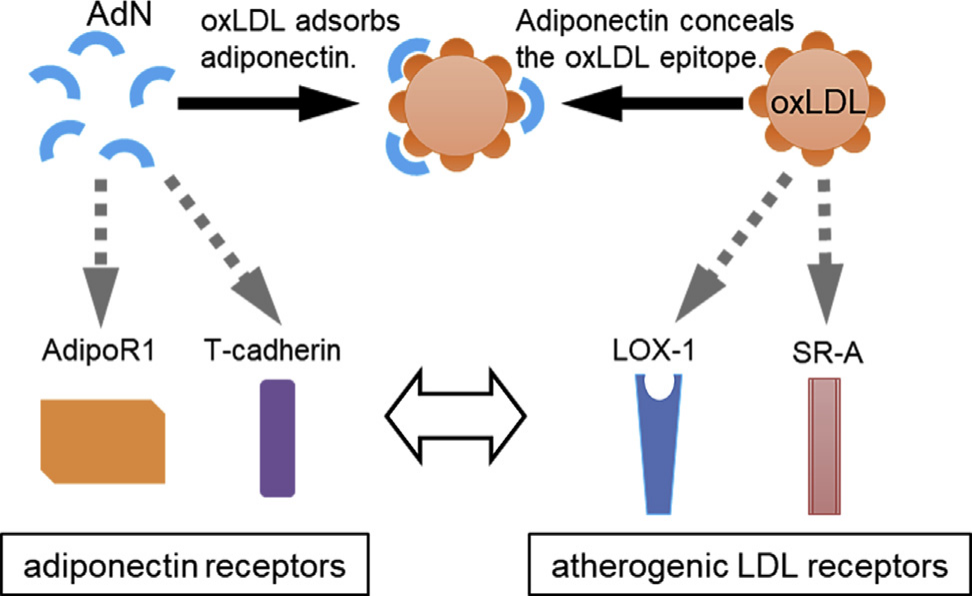
Schematic showing the functional interaction between adiponectin and oxLDL. oxLDL, copper-oxidized low density lipopritein.

In conclusion, we have demonstrated a novel antiatherogenic mechanism of adiponectin by which it directly inhibits atherogenic LDL (Fig. 8). Thus, this study provides a biologically relevant explanation for the well-known properties of adiponectin. Human studies are warranted for further investigating this new aspect of adiponectin biology.

### Data availability

The authors declare that all data supporting the findings of this study are contained within the manuscript.

### Note added in proof

Very recently, while our manuscript was under review at the Journal of Lipid Research, Ye et al. reported in PNAS (29:17381-17388; 2020) that adiponectin binds to anionic phospholipids and sphingolipids.

Addition of any more material should be requested at PNAS.

## Conflict of interest

The authors declare that they have no conflicts of interest with the contents of this article.
